# Establishing an oxidative stress mitochondria-related prognostic model in hepatocellular carcinoma based on multi-omics characteristics and machine learning computational framework

**DOI:** 10.1007/s12672-024-01147-1

**Published:** 2024-07-16

**Authors:** Yitian Wei, Lujuan Ma, Qian Peng, Lin Lu

**Affiliations:** Department of Medical Oncology, the Second Affiliated Hospital, School of Medicine, South China University of Technology, Guangzhou, China

**Keywords:** Mitochondria, Oxidative stress, Hepatocellular carcinoma, Machine learning, Multi-omics analysis

## Abstract

Hepatocellular carcinoma (HCC) has high incidence and mortality rates worldwide. Damaged mitochondria are characterized by the overproduction of reactive oxygen species (ROS), which can promote cancer development. The prognostic value of the interplay between mitochondrial function and oxidative stress in HCC requires further investigation. Gene expression data of HCC samples were collected from The Cancer Genome Atlas (TCGA), Gene Expression Omnibus (GEO) and International Cancer Genome Consortium (ICGC). We screened prognostic oxidative stress mitochondria-related (OSMT) genes at the bulk transcriptome level. Based on multiple machine learning algorithms, we constructed a consensus oxidative stress mitochondria-related signature (OSMTS), which contained 26 genes. In addition, we identified six of these genes as having a suitable prognostic value for OSMTS to reduce the difficulty of clinical application. Univariate and multivariate analyses verified the OSMTS as an independent prognostic factor for overall survival (OS) in HCC patients. The OSMTS-related nomogram demonstrated to be a powerful tool for the clinical diagnosis of HCC. We observed differences in biological function and immune cell infiltration in the tumor microenvironment between the high- and low-risk groups. The highest expression of the OSMTS was detected in hepatocytes at the single-cell transcriptome level. Hepatocytes in the high- and low-risk groups differed significantly in terms of biological function and intercellular communication. Moreover, at the spatial transcriptome level, high expression of OSMTS was mainly in regions enriched in hepatocytes and B cells. Potential drugs targeting specific risk subgroups were identified. Our study revealed that the OSMTS can serve as a promising tool for prognosis prediction and precise intervention in HCC patients.

**Supplementary Information:**

The online version contains supplementary material available at 10.1007/s12672-024-01147-1.

## Introduction

Liver cancer is the fourth leading cause of cancer-related death worldwide, with hepatocellular carcinoma (HCC) being one of its most common types [1]. Surgical resection, percutaneous ablation, radiation therapy, and systemic treatment are among the various treatment options for HCC. However, HCC is often diagnosed at an advanced stage, rendering surgical treatments ineffective. In such cases, systemic treatment with tyrosine kinase inhibitors may be a viable option, although this treatment is beneficial for only 30% of HCC patients and drug resistance may occur during the treatment [2]. Like many other cancers, HCC is characterized by tumor heterogeneity, which often leads to a poor prognosis. Many studies have been conducted to explore more effective models to precisely predict the prognosis of HCC patients. However, there are currently limited prognostic models available for clinical application in such patients [3]. There is an urgent need for biomarker-based models to predict the prognosis of patients with HCC and provide evidence for precision therapy.

Oxidative stress is a state of imbalance between reactive oxygen species (ROS) production and the efficiency of antioxidants [4]. Oxidative stress is closely associated with the occurrence and progression of various physiological diseases, such as cancer, cardiovascular diseases and diabetes mellitus [5]. Previous studies have demonstrated that oxidative stress enhances the risk of developing hepatocarcinogenesis [6]. Hepatocytes are impacted by ROS generated through electron leakage from mitochondrial electron transport, resulting in the activation of carcinogenic pathways [7]. The tumor microenvironment (TME) plays a crucial role in HCC development, coexisting and interacting with various immune cells to support tumor growth [8]. HCC is an inflammation driven tumor, and acute and chronic inflammation can induce oxidative stress in the liver [7]. Immune cells play a crucial role in inhibiting ROS-induced inflammation within the microenvironment [9]. Excessive activation of cell growth, differentiation, and survival signaling pathways due to ROS within TME can promote malignant transformation [10]. Mitochondria are present in almost all eukaryotic cells and play important roles in energy metabolism [11], cell death regulation [12], and hormone signaling [13]. ROS produced by mitochondria affect mitochondrial membrane and the region near the mitochondrial DNA (mtDNA) formation site, leading to mitochondrial dysfunction and further production of ROS [14]. Previous studies have indicated that ROS accumulation and mtDNA alterations affect the signal transduction and mitochondrial biogenesis of cancer cells, thereby promoting the progression of HCC [15]. Specifically, ROS production and abnormal mtDNA repair lead to defects in mitochondrial respiration and adenosine triphosphate production [16]. In addition, due to increased ROS levels, the expression of mitochondrial transcription factor (TFAM) A is downregulated, affecting mtDNA transcription [17].

These features make oxidative stress-related genes and mitochondrial-related genes closely correlated with the prognosis of HCC. However, few studies have examined the association between oxidative stress-related genes and mitochondria-related genes in patients with HCC and explored the potential of this association in the clinical setting. We developed the novel oxidative stress mitochondria-related signature (OSMTS) to predict the prognosis of HCC. In this study, we investigated the characteristics of oxidative stress mitochondria-related genes across multi-omics levels. We utilized bulk transcriptomes to identify such genes and then adopted a new machine learning framework, which included multiple machine learning algorithms, to construct a consensus OSMTS. The mechanism underlying the OSMTS was investigated at the bulk transcriptome, single-cell transcriptome, and spatial transcriptome levels, which revealed close correlations between the OSMTS and the prognosis and immune status of HCC.

## Materials and methods

### Data collection

We obtained RNA sequencing (RNA-seq) data and corresponding clinical information for HCC patients from three databases: the University of California Santa Cruz (UCSC) Xena database (https://xena.ucsc.edu/), the Gene Expression Omnibus (GEO, http://www.ncbi.nlm.nih.gov/geo), and the International Cancer Genome Consortium (ICGC, https://dcc.icgc.org/). Patients with incomplete or missing clinical or pathological data were excluded. The total analysis included 822 samples, with 368 samples from The Cancer Genome Atlas Liver Hepatocellular Carcinoma (TCGA-LIHC) dataset, 242 samples from the GSE14520 dataset, and 212 samples from the Liver Cancer—RIKEN, JP (ICGC-LIRI-JP) dataset. These datasets were preprocessed into numerical feature matrices. To eliminate differences between the samples in each dataset, we used the “normalizeBetweenArrays” function from the “limma” R package. There was not information from the testing data was used for the training data normalization. Additionally, we collected the single-cell RNA sequencing dataset GSE149614 from the GEO database. This dataset included 10 patients with primary or metastatic HCC [18]. Spatial transcriptome data were retrieved from a previous literature [19].

### Identification of prognostic mitochondria-related genes and oxidative stress-related genes

To identify genes associated with mitochondria, we collected 1136 mitochondria-related genes from a previously reported literature [20]. In addition, a list of 1188 oxidative stress-related genes with a relevance score ≥ 7 was downloaded from the GeneCards database.

We utilized univariate Cox regression to investigate potential prognostic mitochondria-related genes and oxidative stress-related genes in HCC patients. Genes with hazard ratios (HRs) > 1 were included for further analysis. In addition, Pearson correlation analysis was performed for prognostic mitochondria-related genes and oxidative stress-related genes in the TCGA-LIHC dataset to identify co-expressed genes with a correlation coefficient (R) greater than 0.6 and *p* value less than 0.001. “Limma” R package was utilized to detect differentially expressed genes (DEGs) between HCC and normal tissues. The inclusion criteria were |log_2_ fold change| (|log_2_FC|) > 1 and an adjusted *p* value < 0.05 [21]. The OSMT genes were identified using the “VennDiagram” R package.

### Functional enrichment analysis

To explore the biological functions and pathway processes associated with OSMT genes, we conducted Kyoto Encyclopedia of Genes and Genomes (KEGG) and Gene Ontology (GO) analyses by using the “ClusterProfiler” package [22, 23]. The above genes were converted to ENTREZID to perform KEGG and GO analyses, and an adjusted *p* value < 0.05 was regarded as a criterion. In addition, Gene Set Enrichment Analysis (GSEA) and Gene Set Variation Analysis (GSVA) were employed to investigate the heterogeneity of various biological processes [24].

### Construction of prognostic signature by integrative machine learning approaches

We utilized OSMT genes to construct a prognostic model with high prediction accuracy. The TCGA-LIHC dataset was used as the training set, GSE14520 dataset and ICGC-LIRI-JP dataset were used as the external validation sets. We used multiple machine learning algorithms, including multilayer perceptron neural network (NN-MLP), logistic regression, linear discriminant analysis, quadratic discriminant analysis (QDA), k-nearest neighbor, decision tree (DT), random forest, XGBoost, ridge regression, least absolute shrinkage and selection operator (LASSO) regression, support vector machine (SVM), gradient boosting, stepwise + logistic regression, and naïve Bayesian methods. We used multiple algorithms in the training dataset, and constructed a model based on a tenfold cross-validation framework. In addition, we utilized a combination of LASSO feature genes and certain machine learning algorithms to calculate whether the model was more accurate after LASSO screening genes. Selecting feature genes can help to eliminate feature redundancy and choose the most informative features. All model constructions, such as LASSO gene screening and tenfold cross-validation, were performed in the training set and then validated in the two external validation sets. The constructed models were evaluated in both the training set and the external validation sets. We ranked the predictive performance of the models and then screened out the algorithms with high rankings. Using machine learning algorithms, we established a final signature for predicting the overall survival (OS) of patients with HCC.

### Survival analysis of the patients training and external validation sets

According to the median OSMT gene risk score, the training set and the external validation sets were divided into high- and low-risk groups. We adopted the “survminer” R package to perform Kaplan–Meier (K-M) curves and determine whether there was a significant difference in overall survival between the high- and low-risk groups. Subsequently, univariate and multivariate Cox regression analyses were utilized to assess whether the OSMTS can be an independent prognostic factor in HCC patients. We explored the relationships between the risk score and several clinical characteristics (age, gender, T stage, M stage, N stage, and tumor stage) in the training set and external validation set. Based on the risk score, we constructed a nomogram to quantify the predictive power of the signature. The decision curve analysis (DCA) function in the “ggDCA” R package was also utilized to evaluate the clinical usefulness of the model at different risk thresholds [25].

### Evaluation of immune cells and immune functions

To identify the differences in immune cell infiltration and immune functions between the high- and low-risk groups, we utilized the CIBERSORT algorithm to quantify the infiltration of 22 immune cell types. Furthermore, we employed the ESTIMATE algorithm to validate the reliability of the CIBERSORT results. The expression of immune checkpoint genes was compared between the two groups. The relationship between the risk score and immune cells was evaluated by Spearman’s correlation.

### Single-cell RNA-seq analysis data collection

The single-cell RNA sequencing data of eight HCC patients were collected from the GSE149614 dataset. We analyzed single-cell sequencing data using the “Seurat” R package [26]. The data were firstly quality-controlled (QC), retaining cells with less than 10% mitochondrial gene content. We selected the cells that expressed 300 genes at least and 4000 at most. The single-cell data were normalized and the first 30 principal components (PCs) were identified via principal component analysis (PCA). We used the “Harmony” R package to eliminate batch effects between samples. The “FindNeighbors” and “FindClusters” functions were utilized to identify cell populations. Furthermore, the t-distributed stochastic neighbor embedding (t-SNE) and Uniform Manifold Approximation and Projection (UMAP) methods were used to visualize the clusters. The “AddModuleScore” function in “Seurat” was used to quantify the expression of OSMT genes in each cell [27]. “CellChat” R package was employed to infer intercellular interactions and to construct a regulatory network based on ligand-receptor crosstalk [28]. We utilized the “netVisual” function to visualize interaction patterns. When evaluating signaling pathways with more than one ligand-receptor pair, we adopted the “netAnalysis_contribution” function to calculate and visualize the contribution of each ligand-receptor pair to the entire signaling pathway.

### Spatial transcriptomic data analysis

Spatial transcriptomic data were obtained from a previous report [19], from which four samples (HCC-1T to HCC-4T; T, tumor section) were selected for our study. We used the “Seurat” R package to analyze and visualize spatial transcriptomic data. The “SCTransform” method was then utilized to normalize the data. To project the spots into a low-dimensional space, we used principal component analysis (PCA) and selected the first 15 principal components (PCs) for downstream processing. After this step, “FindNeighbors” and “FindClusters” functions were adopted to identify the cell clusters. The “AddModuleScore” function was used to quantify the expression of OSMT genes in each cell cluster. “SpatialDimPlot” and “SpatialFeaturePlot” were combined to visualize the cell expression levels in the spatial transcriptome data. To integrate single-cell RNA-seq and spatial transcriptomic data, we used multimodal intersection analysis (MIA) [29], which was conducted by collecting cell type-specific and tissue region-specific genes to calculate whether their overlap was higher (enrichment) or lower (depletion) than that expected by chance.

### Importance of the OSMTS in drug sensitivity

To achieve personalized treatment for patients with HCC, we used the “pRRophetic”R package to predict chemotherapy sensitivity in HCC patients with different risk scores. The patient's tissue gene expression was compared to the expression profile of the cancer cell line, and the half-maximal inhibitory concentration (IC50) was calculated. We used the Wilcoxon test to detect the differences in the IC50 between the high- and low-risk groups, and *p* < 0.05 was considered to indicate statistical significance.

### Statistical analyses

All the statistical analyses were conducted using the R Studio Server (version: 4.3.2). We used false discovery rate-corrected *p* values to evaluate the significant differences in DEGs. Kaplan–Meier (K-M) curves were generated for survival analysis of HCC patients by using the log-rank test. To compare data from categorical variable data between the training and validation datasets, we employed the chi-squared test. Univariate and multivariate Cox regression analyses were further utilized to determine independent predictors of HCC patient survival. Spearman’s correlation analysis was performed to explore the correlation between the risk score and immune cell infiltration. Unless otherwise stated, *p* < 0.05 was regarded as statistically significant. * *p* < 0.05; ** *p* < 0.01; *** *p* < 0.001; **** *p* < 0.0001.

## Results

### Identification of prognostic OSMT genes

**Fig. 1:**
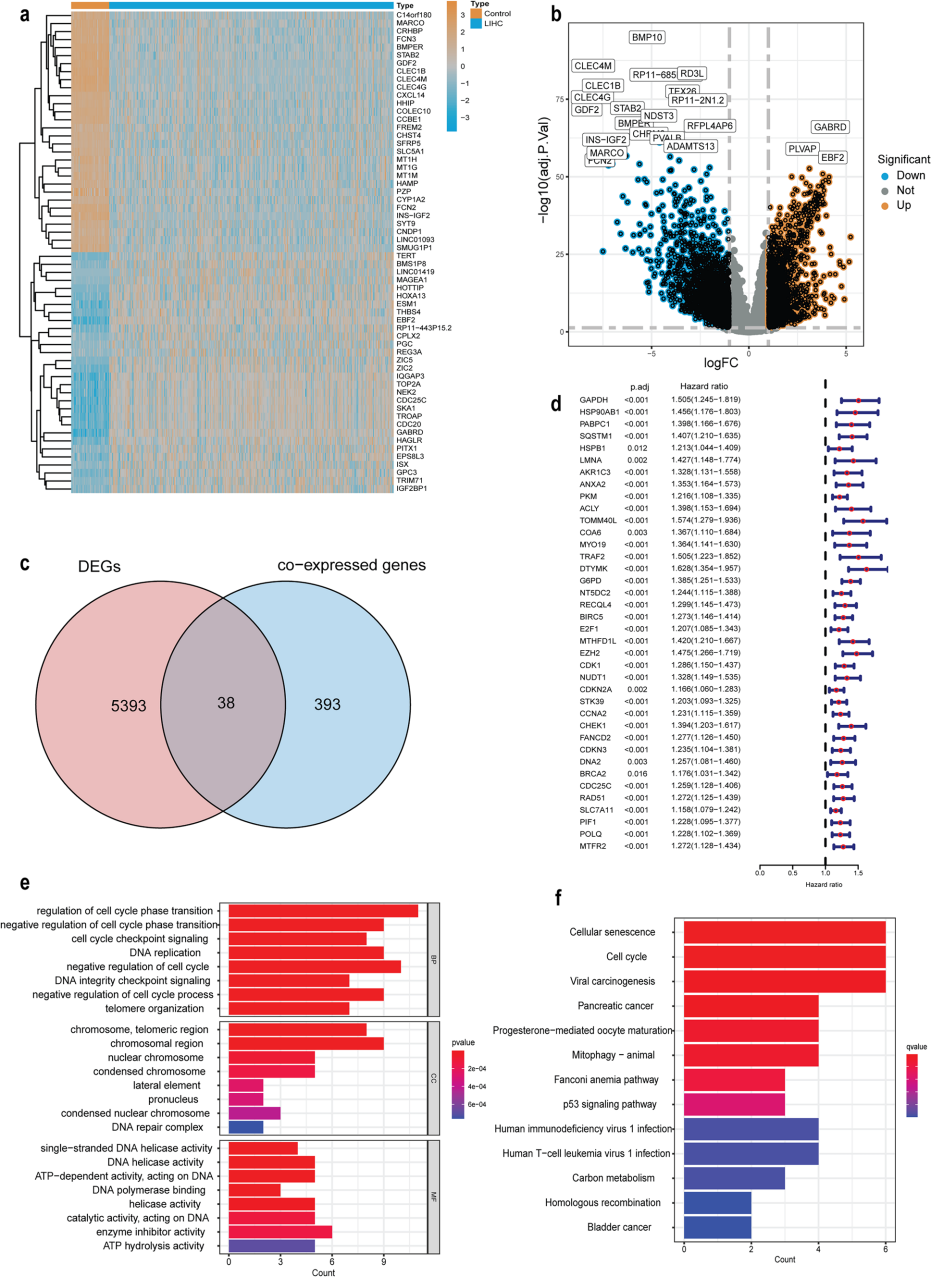
Identification of the OSMT genes in the TCGA dataset. **a** Heatmap shows partially downregulated and upregulated genes between HCC and normal liver tissues (control) in the TCGA-LIHC dataset. **b** The volcano plot for the DEGs. **c** Venn diagram to classify the OSMT genes between DEGS and co-expressed genes. **d** The association between OSMT genes expression and prognosis by forest plot. **e** Barplot of GO analysis of 38 OSMT genes. **f** Barplot of KEGG analysis of 38 OSMT genes

We obtained 424 bulk RNA-seq data from the TCGA database, including 374 tumor samples and 50 normal samples. Among these samples, 5393 differentially expressed genes (DEGs) were identified in HCC patients (Supplementary Table 1, *p* < 0.05 and |log_2_FC|≥ 1). A cluster heatmap and volcano plot were employed to visualize the DEGs (Fig. 1a, b). To identify mitochondria-related genes, we collected 1136 genes from the relevant literature [20]. Additionally, a list of 1188 oxidative stress-related genes with a relevance score ≥ 7 was obtained from the GeneCards Database (Supplementary Table 2). Univariate Cox regression was used to explore the relationship between these genes and HCC patient prognosis. The results showed that 296 mitochondria-related genes and 255 oxidative stress-related genes were associated with the prognosis of HCC patients (Fig. S1a, S2a, *p*.adj < 0.05). Additionally, we conducted Pearson correlation analysis (*r* > 0.6, *p* < 0.001) to identify 431 co-expressed genes. The intersection of DEGs and co-expressed genes was identified using a Venn diagram (Fig. 1c). As shown in Fig. 1d, univariate Cox analysis showed that 38 intersecting genes were associated with the prognosis of patients with HCC (*p*.adj < 0.05). These were designated oxidative stress mitochondria-related (OSMT) genes.

We conducted GO and KEGG analyses of the OSMT genes to explore their potential biological functions (Fig. 1e, f). In the GO enrichment analysis, we found that the most enriched biological processes were related to the cell cycle, DNA replication, and telomere organization. The top three cellular components were chromosome, telomeric region, and DNA repair complex. In terms of molecular functions, we found that DNA helicase activity, ATP-dependent activity, and DNA polymerase binding were enriched. Figure 1f showed the 13 major enriched pathways identified by KEGG analysis, including cellular senescence, the cell cycle, viral carcinogenesis, mitophagy and other KEGG pathways.

### Construction of prognostic signature based on integrative machine learning

**Fig. 2:**
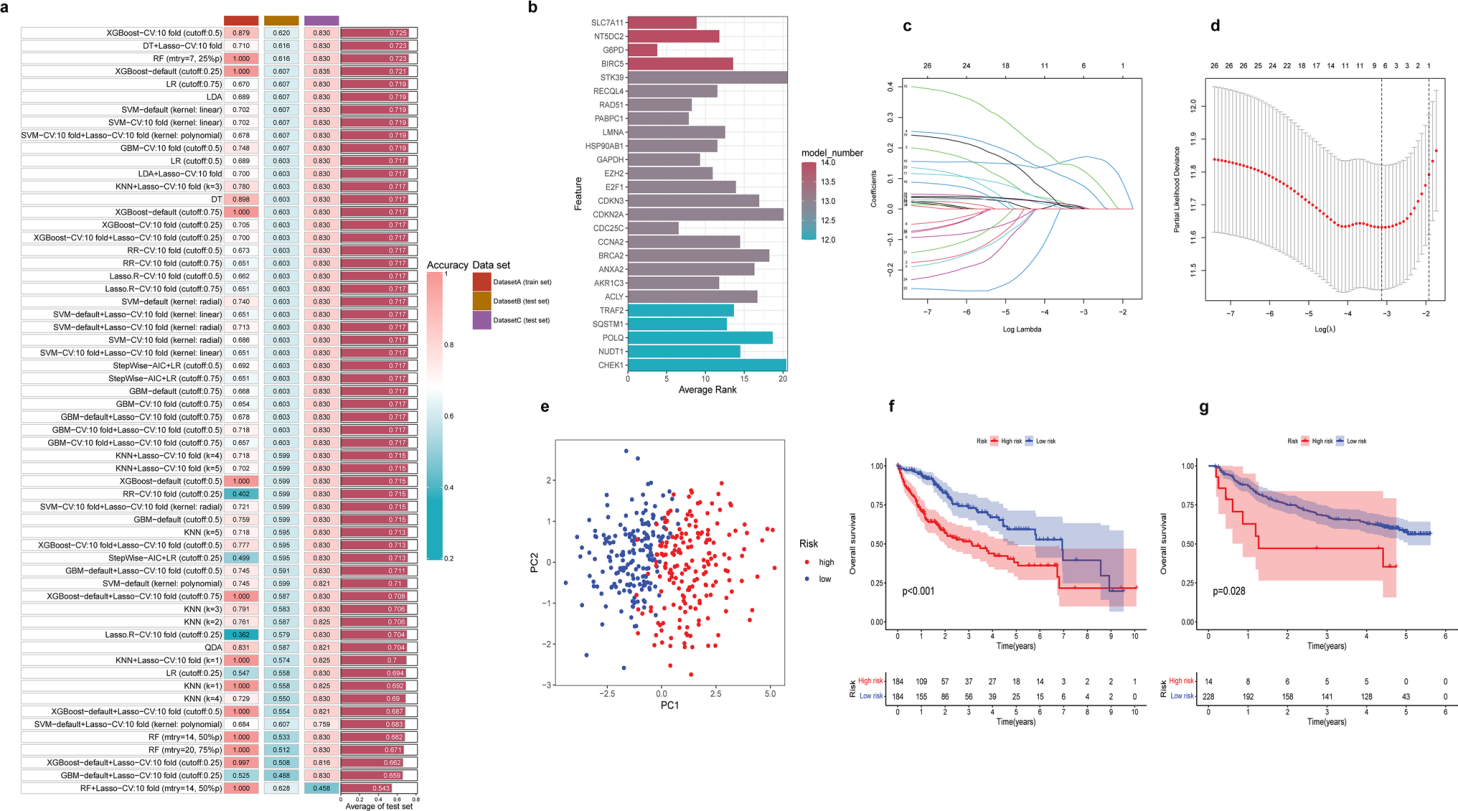
A consensus OSMTS was developed via machine-learning algorithms. **a** The top 60 prediction models were established through machine learning algorithms. **b** Ranking of the importance about 26 OSMT genes. **c** LASSO coefficient profiles of 6 OSMT genes in TCGA-LIHC dataset. **d** LASSO Cox regression identified 6 OSMT genes using the minimum λ. **e** The PCA plots analysis of risk scores. **f**, **g** Kaplan–Meier curve of HCC patients in the TCGA-LIHC dataset (*p* < 0.001) and the GSE14520 dataset (*p* = 0.028)

To construct a consensus OSMTS, we utilized multiple machine learning algorithms to analyze the prognostic genes. The TCGA-LIHC dataset was used as the training set, the GSE14520 and ICGC-LIRI-JP datasets were used as the external validation sets. Among them, there were 26 OSMT genes were identified by these three datasets. These genes included *SLC7A11, G6PD, EZH2, BIRC5, TRAF2, STK39, SQSTM1, RECQL4, RAD51, PABPC1, NUDT1, NT5DC2, LMNA, HSP90AB1, GAPDH, E2F1, CDKN3, CDKN2A, CDC25C, CCNA2, BRCA2, ANXA2, AKR1C3, ACLY, POLQ, and CHEK1*. We fitted the prediction models with a tenfold cross-validation framework in the training set, and the model was then verified in the external validation sets. The algorithms ranked in the top 60 were shown in Fig. 2a. Among all models, XGBoost-CV:tenfold, DT + LASSO-CV:tenfold, GBM-CV:tenfold, SVM-CV:tenfold (kernel: linear), and SVM-default (kernel: linear) algorithms were ultimately used to construct the top five prediction models. These five prediction models performed well in the training set and external validation sets. The genes were ranked by importance in Fig. 2b. The horizontal axis represented the average importance ranking of each gene in the algorithms and the color depth represented the number of genes that appeared. The importance rankings of genes according to each algorithm were provided in Fig. S3a.

The LASSO Cox regression model, which incorporated only six genes, demonstrated suitable predictive ability in the training and external validation sets. We defined these six genes as the suitable prognostic genes for OSMTS. As a result of comprehensive screening, we identified LASSO Cox regression as a predictive model with high accuracy as judged by optimal λ values (Fig. 2c, d). We weighted the expression of the six genes with the coefficients in the LASSO Cox regression model to calculate a risk score for each patient. Based on the median risk scores, we further assigned all patients to high- and low-risk groups. As shown in Fig. 2e, the PCA analysis showed that there were obvious differences between the low- and high-risk groups. In the external validation sets, the performance of the risk model in predicting overall survival was confirmed. Based on the same median risk score used in the training set, GSE14520 was classified into high- and low-risk groups comprising 14 and 228 HCC patients, respectively. The ICGC-LIRI-JP dataset was divided into high- and low-risk groups comprising 4 and 208 HCC patients, respectively, based on the median risk score used in the training set. In both the training set and the external validation sets, patients in the high-risk group exhibited significantly worse overall survival than those in the low-risk group (*p* < 0.05, log-rank test; Fig. 2f, g, and Fig S3b).

### Prognostic value of the OSMTS

**Fig. 3:**
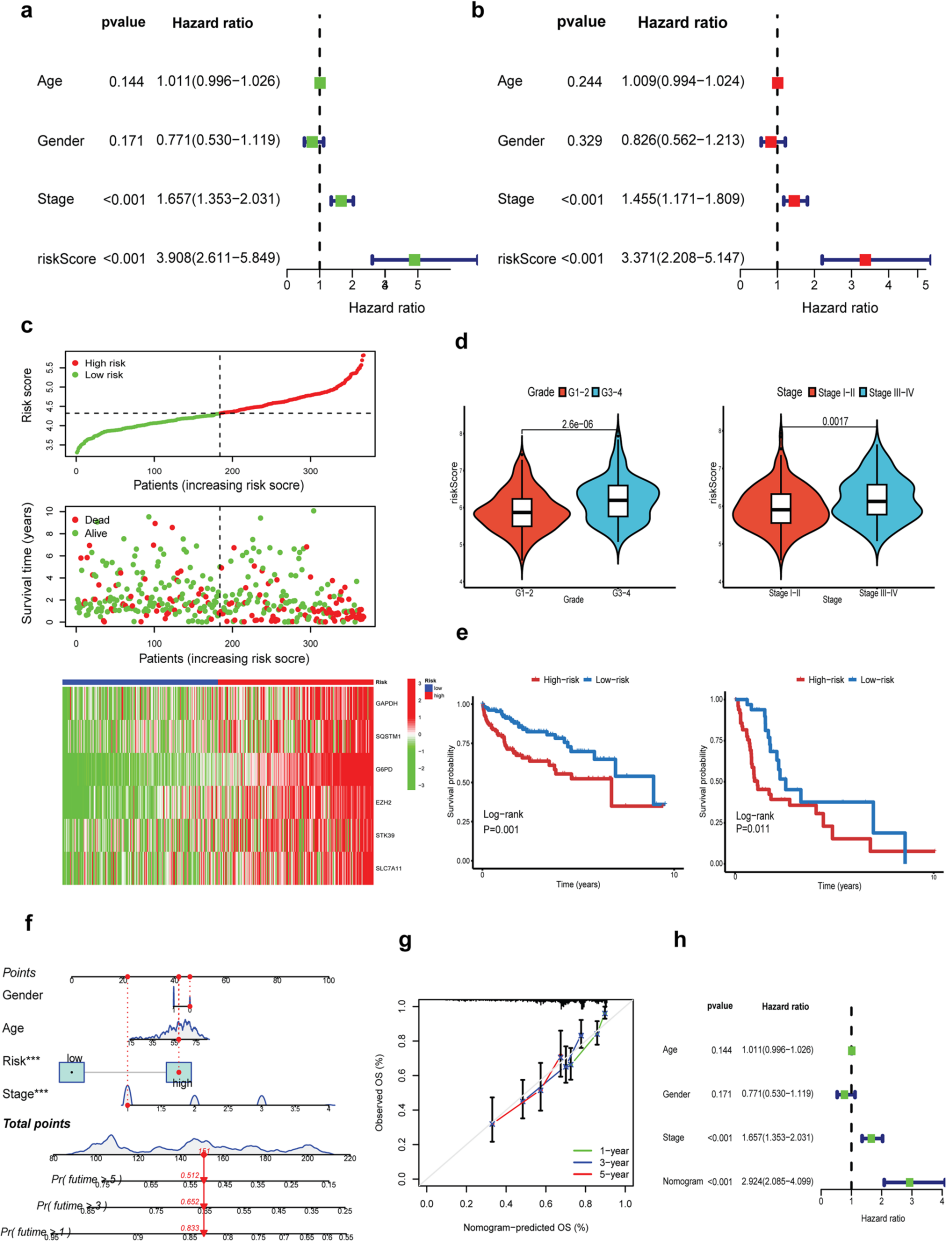
Evaluation of OSMTS and establishment of the nomogram. **a**, **b** Univariate and multivariate Cox analyses of the risk score in TCGA dataset. **c** Risk score values and distribution, OS status and heatmap of the model genes. **d** The difference in risk score between patients grouped by stage, and grade. **e** Kaplan–Meier curves showing stable performance of the risk score in the subgroups of HCC patients, including stage I-II (left) and III-IV (right). **f** Construction of a nomogram to predict the 1-year, 3-year, and 5-year survival probabilities based on pathological features and the model. **g** Calibration curve to verify the efficacy of nomogram for 1, 3, and 5-year OS. **h** Univariate Cox analysis of the clinical characteristics and nomogram for the OS

To determine whether the OSMTS was an independent prognostic factor for HCC, we conducted univariate and multivariate Cox regression analyses on overall survival in the TCGA-LIHC dataset (Fig. 3a, b). Our findings showed that the OSMTS was a significant risk factor for overall survival in the univariate analysis (HR 3.908, CI 2.611–5.849, *p* < 0.001). In the multivariate analysis, OSMTS remained an independent prognostic factor for overall survival (HR 3.371, CI 2.208–5.147, *p* < 0.001), indicating its strong prognostic ability in HCC patients.

Based on the median risk score, all patients in the TCGA-LIHC cohort were separated into the high- or low-risk groups. As the risk score increased, the number of patients who died also increased gradually (Fig. 3c). Moreover, the area under the curve (AUC) value of the ROC curve was 0.781 at 1 year of survival, 0.690 at 3 years, and 0.667 at 5 years (Fig. S3c). We noted that the risk scores were significantly higher in patients with stage III-IV, and G3-4 than in those with stage I-II, and G1-2 (*p* < 0.05, Wilcoxon test) (Fig. 3d). Furthermore, we found that the risk score showed strong prognostic ability in stratified subgroups of clinical features such as stage by K-M curve analysis (Fig. 3e).

With the aim of increasing the clinical applicability of the OSMTS, we developed a nomogram based on the OSMTS to predict the survival probability at 1, 3, and 5 years (Fig. 3f). The calibration plots indicated high consistency between the predicted and actual risk at 1, 3, and 5 years (Fig. 3g). Decision curve analysis (DCA) demonstrated that the nomogram had a greater net clinical benefit than other clinical features (Fig. S3d-f). According to univariate analysis and multivariate analysis, the OSMTS-based nomogram remained an independent prognostic factor for overall survival (univariate analysis: HR 2.924, CI 2.085–4.099, *p* < 0.001; multivariate analysis: HR 2.456, CI 1.364–4.421, *p* < 0.001), indicating that the nomogram was able to predict the overall survival in the TCGA-LIHC cohort (Fig. 3h and Fig. S3g). Overall, these findings showed that the OSMTS-based nomogram was an accurate tool for personalized prognostic prediction in patients with HCC.

### Functional enrichment analysis and assessment of immune status

**Fig. 4:**
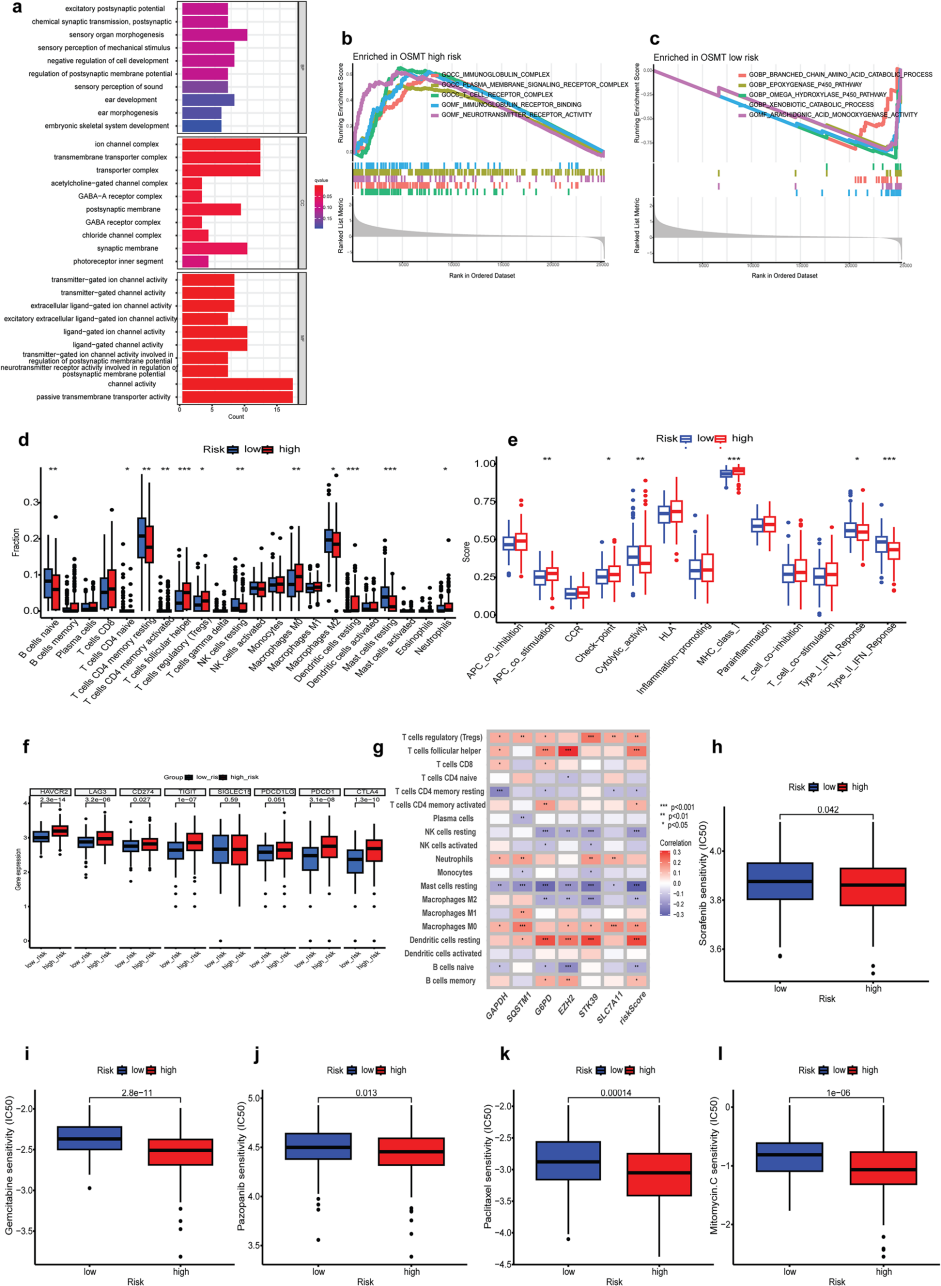
Evaluate the functional enrichment and immune status among high- and low-risk groups. **a** GO analysis used a bar diagram among high- and low-risk groups. **b**, **c** GO enrichment plots from GSEA in the high- and low-risk groups. **d** The scores of 22 immune cells were detected based on high- and low-risk groups. **e** The scores of 13 immune-related functions were determined based on the two groups. **f** Expression levels of immune checkpoint genes in high- and low-risk groups. **g** The correlation of immune cells and 6 OSMTS model genes. Drug sensitivity analyses among high- and low-risk groups. Estimated IC50 of sorafenib (**h**), gemcitabine (**i**), pazopanib (**j**), paclitaxel (**k**), and mitomycin C (**l**)

GO analysis was conducted using 562 DEGs identified between the high- and low-risk groups (*p* < 0.05 and |log_2_FC|> 1). The most highly enriched biological processes were related to chemical synaptic transmission, postsynaptic activity, negative regulation of cell development, and regulation of postsynaptic membrane potential (Fig. 4a). Functional annotations from the GO analysis were further validated and complemented by GSEA. GO enrichment analysis of the high-risk group revealed that the following cellular components were the most enriched: immunoglobulin complex, T-cell receptor complex, and plasma membrane signaling receptor complex (Fig. 4b). In contrast, branched-chain amino acid catabolic processes and the epoxygenase P450 pathway were the most enriched pathways in the low-risk group (Fig. 4c). Differences in the differentially enriched KEGG pathways between the low- and high-risk groups were analyzed by GSVA (Fig. S5a). The PI3K/AKT/mTOR signaling pathway, p53 signaling pathway, spliceosome pathway and RNA degradation pathway were enriched in the high-risk group.

The cellular components of the high-risk groups were closely related to immune cells in the GSEA. To further analyze the differences in specific immune cell infiltration between the high- and low-risk groups, we used the CIBERSORT algorithm to quantify the abundance of infiltrating immune cells in each sample. As shown in Fig. 4d and e, the 12 immune cell types and 6 immune functions exhibited significant differences between the two groups in the TCGA cohort. The proportions of M0 macrophages, T cells regulatory (Tregs) and CD4 + T cell memory activated cells were highly expressed in the high-risk group. Compared with those in the immune microenvironment of the high-risk group, macrophage M2 cells, CD4 + T cell memory resting cells, and mast resting cells were more abundant in the low-risk group and had no anticancer activity. High-risk patients showed higher expression of most major histocompatibility (MHC) class I genes. Recent reports have indicated that MHC class I gene expression was related to IFN-γ and immunosuppressive functions [30]. Compared with those in the high-risk group, the expression of the Type I IFN response and Type II IFN response were upregulated in the low-risk group. Furthermore, the two groups were further compared in terms of immune checkpoint gene expression (*HAVCR2, LAG3, CD274, TIGIT, SIGLEC15, PDCD1LG2, PDCD1, CTLA4*; Fig. 4f). Previous studies have shown that higher expression of immune checkpoint genes was associated with a better response to immune checkpoint inhibitor (ICI) therapy. As shown in Fig. 4f, the expression of immune checkpoint genes, except for *SIGLEC15* and *PDCD1LG2*, was significantly elevated in the high-risk group (*p* < 0.05). The expression of these immune checkpoint molecules was positively correlated with the risk score. Figure 4g revealed the correlation between genes in the OSMTS and immune cell infiltration. We further analyzed the correlation between the OSMTS and immune cells to determine the role of OSMTS in the tumor immune microenvironment (Fig. S4a-h).

Due to the highly dynamic and heterogeneous tumor microenvironment, drug resistance was a common problem. We compared the IC50 values of several chemotherapeutic agents between the two groups by Wilcoxon signed-rank test. A low IC50 indicates sensitivity. Patients at high risk had lower IC50 values for sorafenib (*p* = 0.042), gemcitabine (*p* < 0.0001), pazopanib (*p* = 0.013), paclitaxel (*p* < 0.0001), and mitomycin C (*p* < 0.0001). The above chemotherapy may benefit high-risk patients (Fig. 4h-l).

### The OSMTS in the single-cell transcriptome

**Fig. 5:**
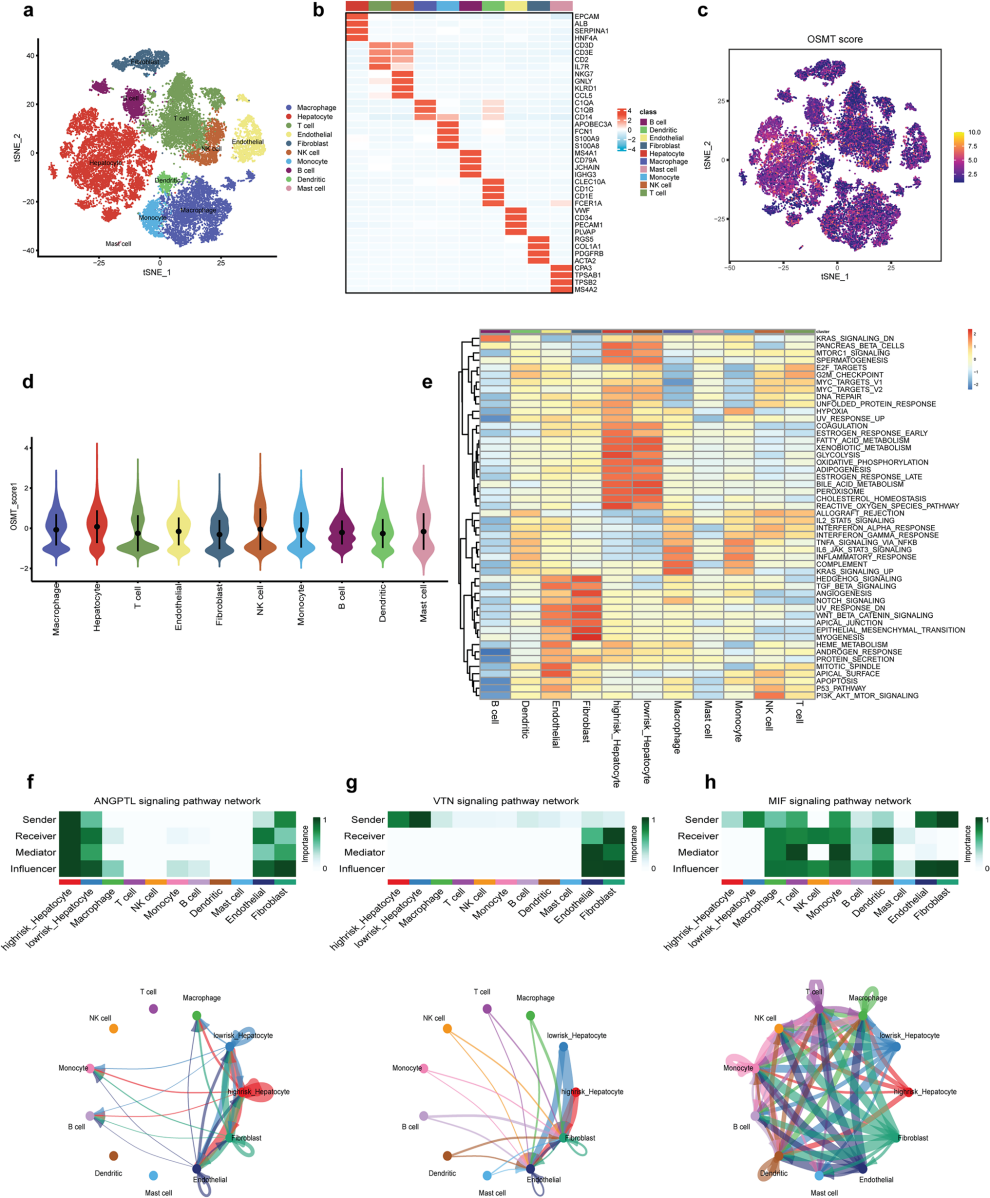
The correlation of OSMTS with single-cell characteristics. **a** t-SNE plot showing the cell types identified by marker genes. **b** Heatmap showing the marker genes in each cell clusters. **c** The distribution of the OSMT score in different cell types. **d** The activity score of OSMT in each cell clusters. **e** GSEA enrichment analysis in high- and low-risk hepatocytes and other cell types. **f–h** Heatmaps showing the roles of different cell types playing in the ANGPTL, VTN, and MIF pathway networks. Circles plots showing the signaling pathway networks

We obtained single-cell RNA-seq data of eight HCC patients in the GSE149614 dataset [18]. We utilized the Harmony package to remove batch effects. Subsequently, principal component analysis (PCA), t-distributed stochastic neighbor embedding (t-SNE), and Uniform Manifold Approximation and Projection (UMAP) were then applied to the top 2000 variant genes for dimensionality reduction. We clustered all cells into 23 clusters with a resolution of 0.6. Using marker genes for different cell types, we annotated the 10 major clusters based on the marker genes of different cell types, namely, hepatocytes, T cells, fibroblasts, macrophages, dendritic cells, monocytes, mast cells, endothelial cells, NK cells, and B cells (Fig. 5a), and displayed the marker genes for each cell population in a heatmap (Fig. 5b). To quantify the expression of OSMT genes in different cell types, we used the “AddModuleScore” function in the Seurat package to calculate the expression levels of the 26 genes related to OSMT across all cells (Fig. 5c). Among the 10 cell types, hepatocytes, and NK cells exhibited significantly increased OSMT expression (Fig. 5d). Based on OSMT expression, we classified hepatocytes into high- and low-risk score groups, and identified 5165 DEGs between the two groups for further analyses. GSEA enrichment analysis was used to inform the functions of the high- and low-risk hepatocytes and other cell types. We found that oxidative phosphorylation, peroxisome, and fatty acid metabolism were enriched in the hepatocytes of the low-risk group, whereas the ROS pathway, glycolysis, and hypoxia were enriched in the hepatocytes of the high-risk group (Fig. 5e). Next, we conducted GO and KEGG enrichment analyses to investigate the functional differences in DEGs between these two groups (Fig. S5b, c). GO analysis of the hepatocytes in the high-risk group revealed that the following biological processes were the most enriched: cadherin binding, DNA-binding transcription factor binding and ubiquitin-protein transferase activity.

Next, we investigated the interactions of hepatocytes in the high- and low-risk groups with other types of cells in the TME. We found that hepatocytes with different OSMT scores had diverse communication patterns (Fig. S5d, e). Each cell cluster exhibited different pathways of communication in afferent and efferent modes under high- and low-risk conditions (Fig. S5f, g). Different cell types in the TME can act as senders, receivers, mediators, and influencers in cellular communication (Fig. S6a). Our findings showed that hepatocytes with a high-risk score acted as stronger senders, receivers, mediators and influencers of ANGPTL signaling (Fig. 5f), which was related to vascular growth and tumor cell invasion [31]. Hepatocytes with a low-risk score communicated with more types of TME cells; for instance, these cells acted as senders and influencers in the MIF and VTN signaling pathways (Fig. 5g, h), which were related to immune regulation, cell adhesion and migration. These hepatocytes may be involved in integrin signaling pathways [32]. Among them, the VTN signaling pathway receptors were mainly mediated by the binding of ITGAV and ITGB1 (Fig. S6b). The effects of MIF were mediated by binding to the receptors CD74 and CXCR4, which were involved in MIF cell signaling (Fig. S6c).

To further explore the heterogeneity of hepatocytes in eight HCC patients, we applied type specific markers that categorized 8141 hepatocytes into 13 clusters with a resolution of 0.6. The hepatocyte spots were divided into hepatocytes, inflammatory hepatocytes, malignant hepatocytes, proliferating malignant hepatocytes, and bipotent cells (Fig. S6d) [33, 34]. To quantify the activity of OSMTS in different cell types, we used the "AddModuleScore" to calculate the expression level of OSMTS across all cells. The result showed that OSMT activity was significantly increased in malignant hepatocytes, and bipotent cells (Fig. S6e). This phenomenon may indicate that OSMTS was closely related to the occurrence and development of HCC.

### OSMTS in spatial transcriptome

**Fig. 6:**
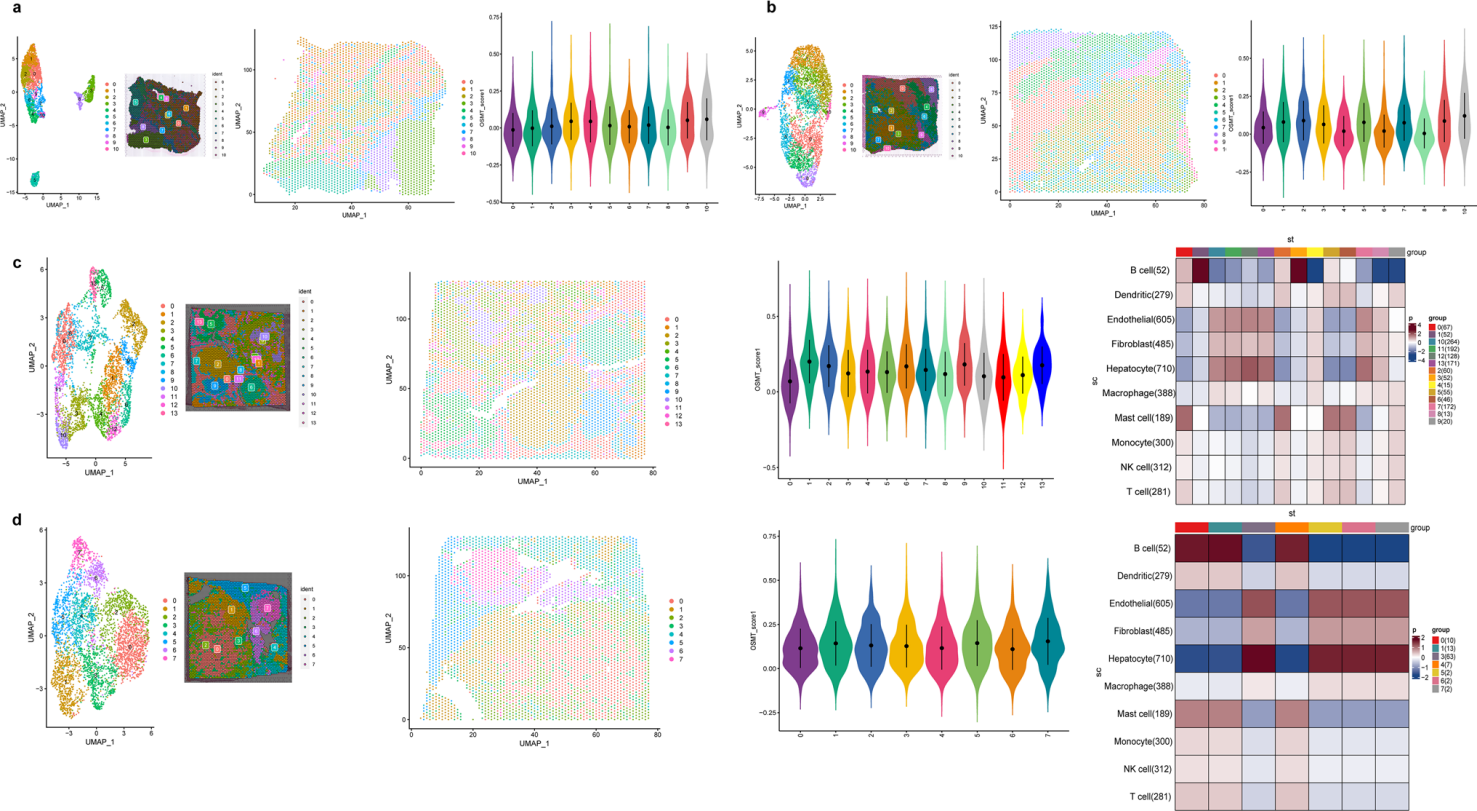
The correlation of OSMTS with spatial transcriptome characteristics. Left: UMAP plot of cell clusters, Middle: UMAP spot of each clusters on H&E staining sections, Right: the distribution of the OSMT score in different cell clusters: **a** HCC-1T; **b** HCC-3T. Left: UMAP spot of cell clusters, Middle: UMAP spot of each clusters on H&E staining sections and the distribution of the OSMT score in different cell clusters, Right: MIA map of the scRNA-seq identified cell types and ST-defined regions: **c** HCC-2T; **d** HCC-4T

Spatial transcriptome data for four samples (HCC-1T, HCC-2T, HCC-3T, and HCC-4T; T, tumor section) were obtained from a previous literature [19]. We performed cluster analysis on these samples to characterize the spatial diversity of HCC. The distribution of the clusters was determined in both the UMAP and tissue physical space. Clusters in HCC-3T and HCC-4T cells were clearly separated, whereas clusters in HCC-1T and HCC-2 T cells overlapped. To quantify the expression of OSMT genes in different cell clusters, the “AddModuleScore” function in the “Seurat” package was used to calculate the expression levels of the 6 gene sets in the model related to the OSMT genes across all clusters. We used the “MIA” algorithm to integrate the single-cell RNA-seq and spatial transcriptomic datasets. As shown in Fig. 6a and b, we found little difference in the OSMT scores in HCC-1T and HCC-3T groups, indicating that OSMT genes were widely expressed in these samples. Among the 13 cell clusters in HCC-2T cells, Clusters 1 and 13 had significantly greater OSMT scores (Fig. 6c). The “MIA” algorithm further revealed that B cells were enriched in Cluster 1. As expected, hepatocytes were enriched in Cluster 13, which may indicate that these cells were dispersed in the tumor sections. Unlike the overlapping characteristics of the HCC-2T clusters, the HCC-4T clusters exhibited a clear separation. As shown in Fig. 6d, HCC-4T patients in Clusters 1, 5 and 7 had higher OSMT scores. In Cluster 1, B cells were enriched, as indicated by the “MIA” algorithm. Hepatocytes were enriched in Clusters 5 and 7, which may indicate that these cells were enriched at the edge of the tumor section.

## Discussion

Recent studies have shown that ROS play important roles in immune responses [35, 36]. Specifically, the concentrations of ROS can impact the function of immune cells within the TME, impairing their ability to effectively exert their antitumor effects and leading to resistance to certain types of immunotherapies. Thus, targeting oxidative stress in the TME may potentiate the efficacy of tumor immunotherapy. Many studies have demonstrated the close association between oxidative stress and mitochondria, and mitochondrion-targeted antioxidants have been found to be effective in cancer prevention and anticancer therapy [37]. These findings indicate close relationships among oxidative stress, mitochondria, and antitumor immunity. Therefore, the OSMTS may be useful as a personalized indicator for predicting patient prognosis and evaluating treatment response.

In recent years, several studies have aimed to establish gene signatures to better understand the prognostic classification of HCC. For example, Ren et al. [3] reported an oxidative stress-related prognostic signature that predicted overall survival in HCC. Shi et al. [20] developed a prognostic signature based on mitochondria-related genes to predict prognosis in HCC. As well as Zhang et al. [38] constructed a prognostic model based on circRNA-mediated competing endogenous RNA to validate the prognosis of HCC, and all of these studies showed a certain degree of predictive ability for the prognosis of HCC patients. These signatures were mainly constructed at the bulk RNA level. Our model was validated at bulk RNA level, single-cell level, and spatial transcription level. The optimal model was screened through multiple machine learning algorithms.

In our study, we constructed a novel prognostic OSMTS that represents a promising tool for prognostic prediction and prevention in patients with HCC. To our knowledge, this is the first prognostic marker constructed by combining mitochondria-related genes and oxidative stress-related genes in HCC patients. In contrast to previous studies, we investigated the molecular mechanisms of HCC development and progression from the perspectives of genomics, bulk transcriptomics, and single-cell transcriptomics, and we added spatial transcriptomics to the analysis for the first time. This signature will aid in accurately predicting the prognosis of HCC patients and provide new insights for HCC immunotherapy. In summary, this signature has enormous potential as a novel biomarker for HCC.

We utilized bulk transcriptomes to identify oxidative stress- and mitochondria-related genes. We collected expression data from 822 samples, with 368 samples from the TCGA-LIHC dataset, 242 from the GSE14520 dataset and 212 from the ICGC-LIRI-JP dataset. Subsequently, we employed a novel machine learning framework to construct a consensus OSMTS. The signature depended on the expression patterns of 26 genes, specifically *SLC7A11, G6PD, EZH2, BIRC5, TRAF2, STK39, SQSTM1, RECQL4, RAD51, PABPC1, NUDT1, NT5DC2, LMNA, HSP90AB1, GAPDH, E2F1, CDKN3, CDKN2A, CDC25C, CCNA2, BRCA2, ANXA2, AKR1C3, ACLY, POLQ, and CHEK1*. Among them, the LASSO-screened genes, which included six genes (*SQSTM1, EZH2, STK39, SLC7A11, GAPDH, and G6PD*), showed high predictive value. Therefore, we defined these six genes as having a suitable prognostic value for OSMTS to reduce the difficulty of clinical application. Many of these genes have been reported to be involved in the development of HCC. SQSTM1 can activate certain signaling pathways, such as the NF-κB and Nrf2 pathways, which played key roles in the anti-inflammatory response and antioxidant stress response. Aberrant activation of these pathways has been implicated in the carcinogenesis and progression of HCC [39, 40]. High SLC7A11 expression may enhance the ability of tumor cells to defend against oxidative stress and promote tumor growth and chemotherapy resistance [41, 42]. STK39 promoted the growth and metastasis of HCC by activating the PLK1/ERK signal transduction axis [43]. Overexpression of EZH2 has been observed in many types of cancer and is often associated with poor prognosis. Previous studies have demonstrated that EZH2 also plays a role in 5-FU and sorafenib resistance in HCC chemotherapy [44].

Our analysis demonstrated that the OSMTS had high predictive accuracy and high potential for clinical application. Second, we utilized the OSMTS to stratify the risk of HCC patients, and we analyzed their overall survival and clinical characteristics. Based on the OSMTS, the patients were divided into high- and low-risk groups in both the training set and external validation sets. Patients in the high-risk group had worse overall survival than did those in the low-risk group. The risk score was associated with clinicopathological characteristics and was shown to be an independent prognostic factor in patients with HCC. Identifying individuals at high risk of developing HCC and implementing cost-effective, targeted prevention strategies are of utmost importance [45]. In our study, machine learning algorithms were utilized to stratify patients with HCC according to their risk level. This strategy can enable oncologists to develop personalized intervention strategies for patients at each risk level. For individuals identified as high risk, oncologists should recommend more frequent check-ups and provide more detailed health instructions [27].

Nomogram and calibration analyses demonstrated the excellent clinical predictive performance of the model. GO analysis and GSEA revealed that the high-risk group had the greatest enrichment of the immunoglobulin complex and T-cell receptor complex. The differences in the KEGG pathways between the high- and low-risk groups were analyzed by GSVA. The PI3K/AKT/mTOR signaling pathway, p53 signaling pathway, spliceosome pathway and RNA degradation pathway were enriched in the high-risk group. Previous studies have reported that these pathways are abnormally overactivated in various types of cancer. Agents that target these aberrantly hyperactivated pathways may have the potential to inhibit the progression of HCC. Dysregulated activation of the PI3K/AKT/mTOR signaling pathway affects a wide range of processes, including cell proliferation, metabolism, tumor cell differentiation, autophagy, and epithelial–mesenchymal transition (EMT). Importantly, the PI3K/AKT/mTOR pathway is aberrantly activated in nearly 50% of patients with HCC [46]. Previous studies have demonstrated that the P53 signaling pathway is closely related to increased mitochondrial fission and elevated ROS production [47]. Additionally, analysis of immune cell infiltration indicated that T cells, B cells, macrophages, and dendritic cells were significantly associated with the risk score. High-risk patients had higher expression of the majority of immune checkpoint genes, suggesting that the expression of these genes was positively correlated with the risk score. HCC is relatively insensitive to chemotherapeutic agents due to resistance and heterogeneity. The response to several chemotherapy drugs differed between the two groups, suggesting that the OSMTS could also be useful for selecting chemotherapy drugs for HCC patients.

Based on multi-omics data, we elucidated the molecular basis and potential mechanism of this signature, confirming that the OSMTS was closely related to the development and prognosis of HCC. At the single-cell transcriptome level, OSMTS showed the highest expression in hepatocytes. GO analysis revealed enrichment of hepatocytes in the high-risk group in biological processes such as glycolysis and hypoxia. Changes in energy metabolism are among the "hallmarks of cancer". This metabolic phenotype is characterized by a preference for glycolysis to produce energy in an oxygen-independent manner. In terms of intercellular communication, hepatocytes in the low-risk group communicate with more types of TME cells. Hepatocytes in the high-risk group act as senders, receivers, mediators, and influencers of ANGPTL signaling. ANGPTLs are a family of proteins whose structures are similar to those of ANGs [48]. ANGPTLs were reported to participate in cancer metastasis by regulating angiogenesis. The high expression of ANGPTLs in patients with colorectal cancer (CRC) may be associated with a relatively poor prognosis. Previous experiments have shown that ANGPTL4 promotes the proliferation and migration of CRC cells [49]. High expression of ANGPTL pathway members in the hepatocytes in the high-risk group may be a potential therapeutic target for patients with HCC in the future. At the spatial transcriptome level, the OSMTS was widely enriched in tumor samples, particularly in regions with hepatocytes and B-cell enrichment. These regions were widely distributed in the tumor samples. This phenomenon may be due to the diffuse distribution of hepatocytes in the tumor samples. B cells are important regulators of the HCC microenvironment. Some studies have shown that functional mitochondria are indispensable for the differentiation and effector function of B cells [50]. However, excessive mitochondrial mtROS synthesis may inhibit B-cell activation and the differentiation of B cells into antibody-producing plasmablasts [51]. Agents that target B cells may potentially inhibit the progression of HCC.

Recently, noninvasive diagnostic techniques have received increasing attention in the field of cancer research. Tumor tissue obtained through invasive liver biopsies can be used for genetic sequencing, but such invasive procedures carry risks for patients. As an alternative, researchers have begun exploring the use of blood samples from patients. This method is noninvasive, and by analyzing circulating tumor DNA (ctDNA) or circulating tumor cells (CTCs) present in the blood, researchers can identify specific genetic mutations or alterations that characterize tumors. The OSMTS was associated with tumor progression, treatment resistance, and overall prognosis. By analyzing the levels of OSMT genes in blood cells, clinicians can make more accurate predictions about a patient’s prognosis and response to treatment.

Although our research has produced promising findings, two main limitations must be acknowledged. First, although we evaluated and validated the OSMTS in both the training and external validation datasets, additional samples are required for further validation. Additionally, conducting large-scale and multicenter prospective studies will provide stronger evidence for our findings. Second, additional in vitro and in vivo studies are necessary to investigate the biological functions of OSMT genes in HCC.

## Conclusion

In this study, we constructed the OSMTS as a promising tool for prognosis prediction and prevention in patients with HCC. We used machine learning algorithms to classify patients with HCC according to their risk levels. In addition, we provided novel insights into the molecular mechanisms underlying the development and progression of HCC from the perspectives of bulk transcriptomics, single-cell transcriptomics, and spatial transcriptomics.

### Supplementary Information

Supplementary File 1.

Supplementary File 2.

Supplementary File 3.

## Data Availability

All data are available in a public, open access repository. The data achieved and analyzed in this study are available in the UCSC Xena database (https://xena.ucsc.edu/), Gene Expression Omnibus (GEO, https://www.ncbi.nlm.nih.gov/geo/), and the International Cancer Genome Consortium (ICGC, https://dcc.icgc.org/). Further inquiries can be directed to the corresponding authors.
