# Supplementary material for: Establishing an oxidative stress mitochondria-related prognostic model in hepatocellular carcinoma based on multi-omics characteristics and machine learning computational framework
Source: Discov Oncol. 2024 Jul 16;15:287. doi: 10.1007/s12672-024-01147-1 (PMC11252104; doi:10.1007/s12672-024-01147-1)
Supplement: Supplementary file 1 [file 12672_2024_1147_MOESM1_ESM.docx]

**Establishing an oxidative stress mitochondria-related prognostic model in hepatocellular carcinoma based on multi-omics characteristics and machine learning computational framework**

Yitian Wei ^#^, Lujuan Ma ^#^, Qian Peng ^#^, Lin Lu ^*^

Table of contents

[Supplementary figures 2](#_Toc64552259)

[Supplementary table legends 1](#_Toc64552260)0

**Supplementary figures**

**
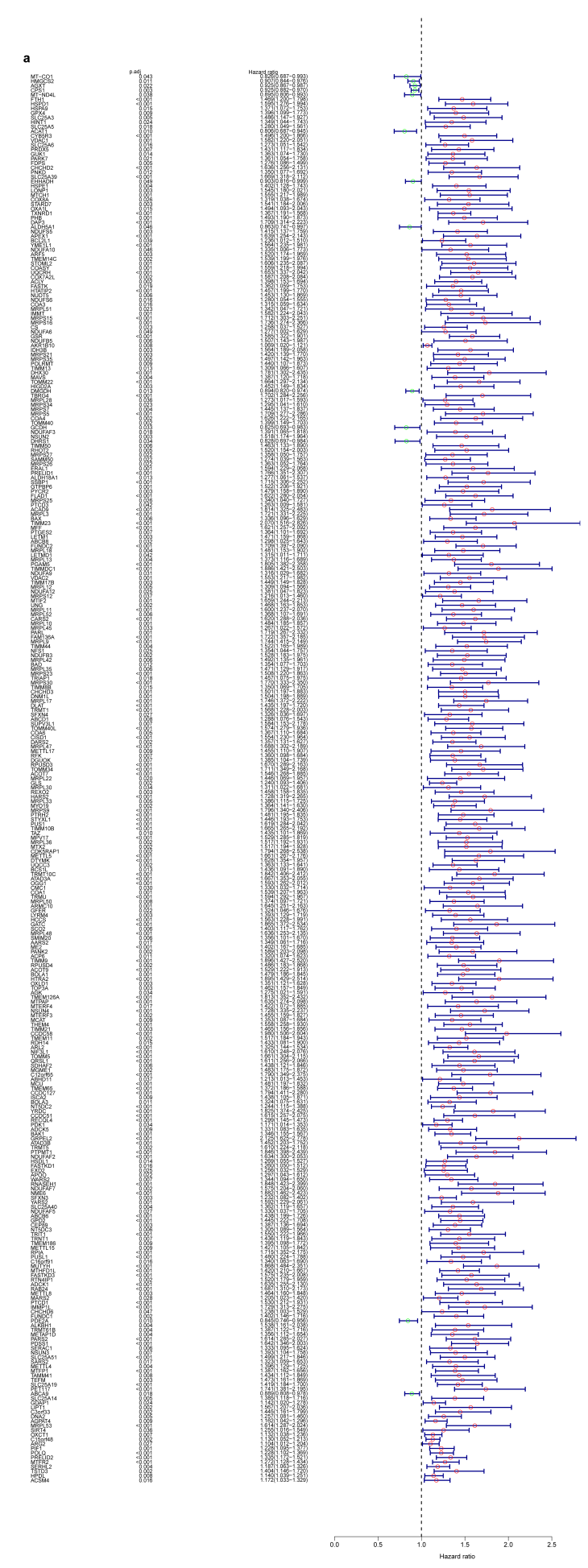
**

**Fig. S1**  The association between mitochondria-related genes expression and prognosis by forest plot.


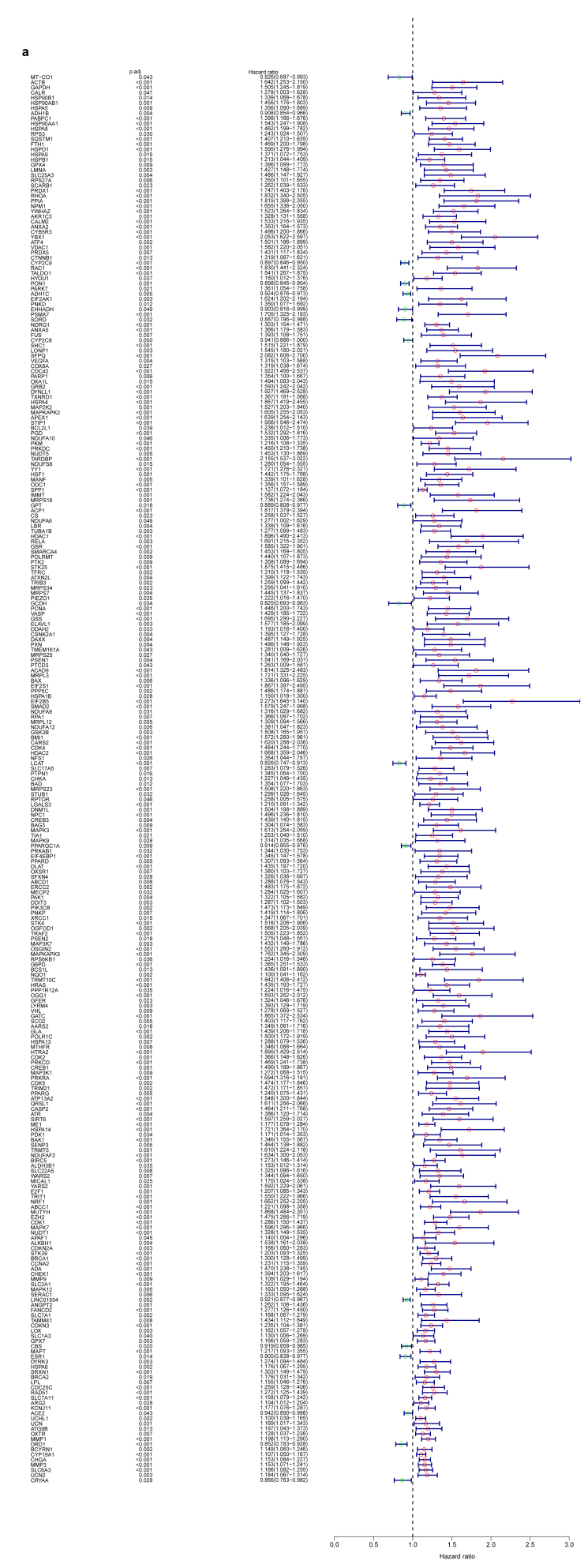


**Fig. S2** The association between oxidative stress-related genes expression and prognosis by forest plot.


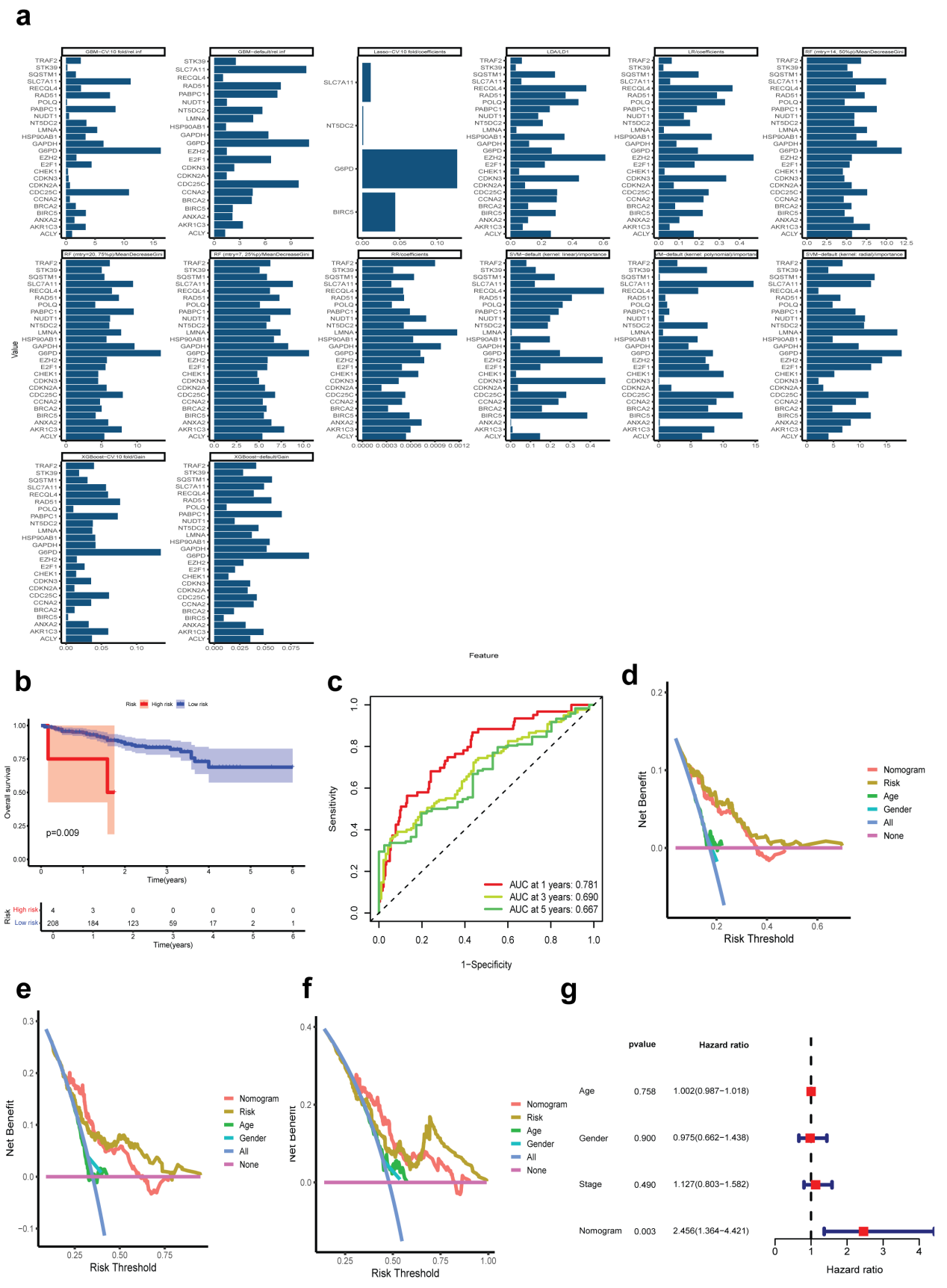


**Fig. S3** The importance rankings of genes and evaluation of OSMTS. **(a)** The importance rankings of genes in each machine learning algorithm. **(b)** Kaplan-Meier curve of HCC patients in the ICGC-LIRI-JP dataset (*p*=0.009). **(c)** ROC curves showing the specificity and sensitivity of OSMTS in predicting 1, 3, and 5-year OS in the TCGA training set. **(d-f)** DCA showing the net benefit by applying the nomogram and other clinical characteristics in 1, 3, and 5-year OS in the TCGA training set. **(g)** Multivariate Cox analysis of the clinical characteristics and nomogram for the OS.


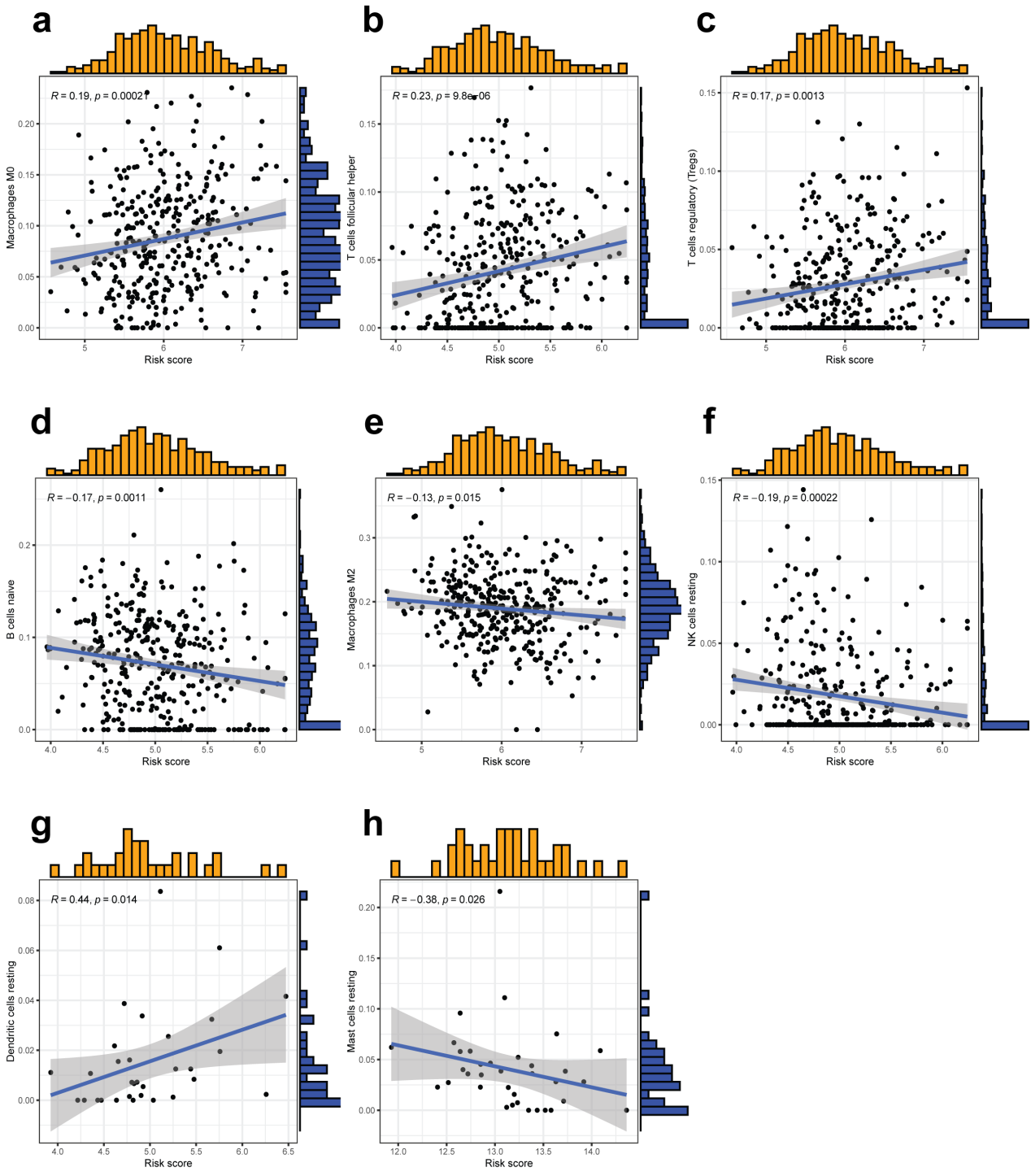


**Fig. S4** Relevance of risk scores for different immune cell types **(a-f)** Relevance of risk scores to macrophages M0, T cells follicular helper, T cells regulatory (Tregs), B cells naive, macrophages M2, and NK cells resting in TCGA dataset. **(g)** Relevance of risk scores to dendritic cells resting in ICGC dataset. **(h)** Relevance of risk scores to mast cells resting in GSE14520 dataset.


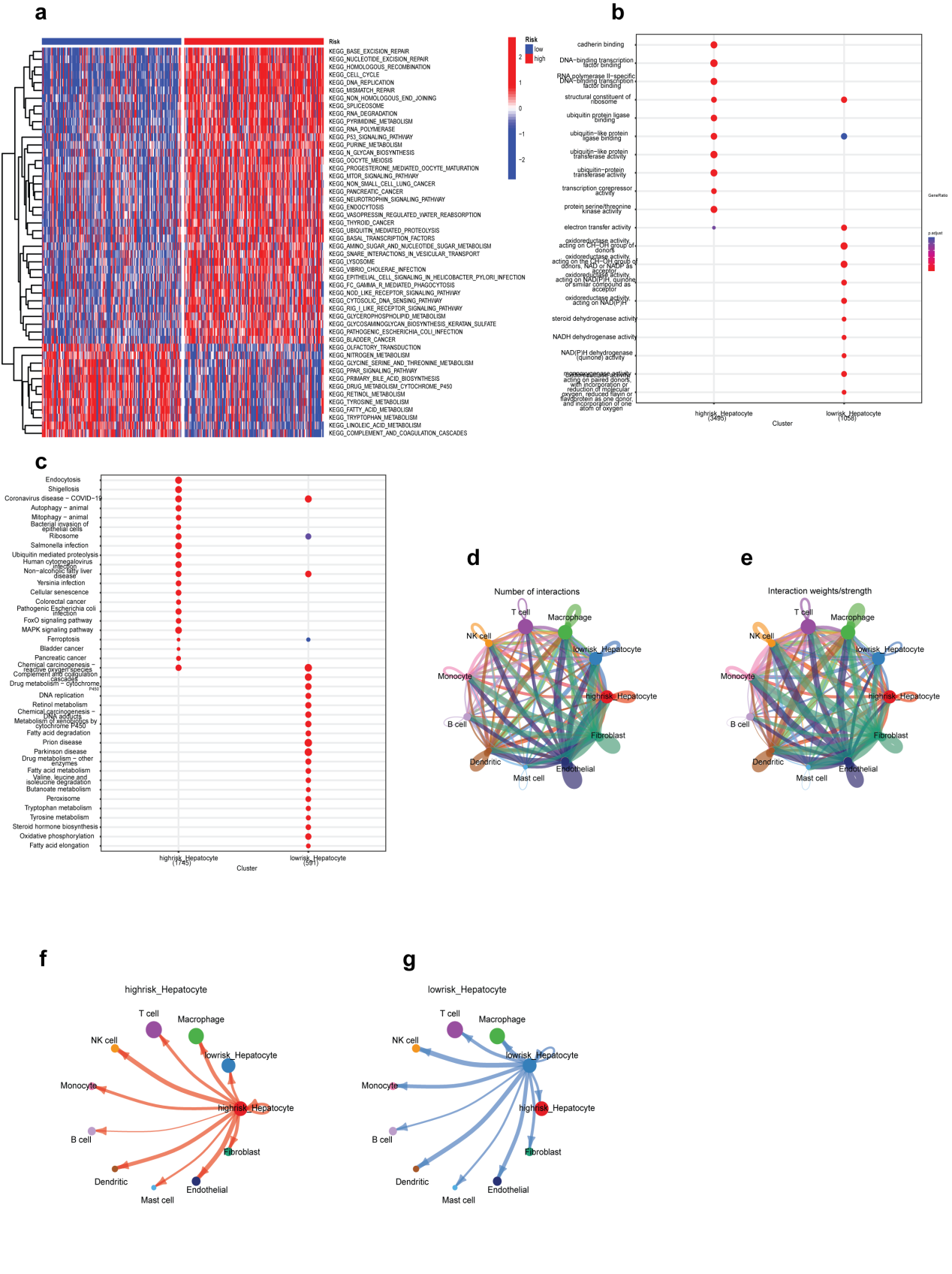


**Fig. S5** The characteristics of OSMTS in single-cell transcriptome level **(a)** Differences in the KEGG pathways between the low- and high-risk groups were analyzed by GSVA. **(b)** GO enrichment analysis of high-and low-risk hepatocytes. **(c)** KEGG enrichment analysis of high-and low-risk hepatocytes. **(d, e)** Circle plots showing the numbers and strengths of interactions, in which each chord diagram represents the ligand-receptor pairs between the two cell types. The round loops with cell type represent interactions within the same cell type. **(f)**The ligand-receptor interactions sent from high-risk hepatocytes. **(g)** The ligand-receptor interactions sent from low-risk hepatocytes.


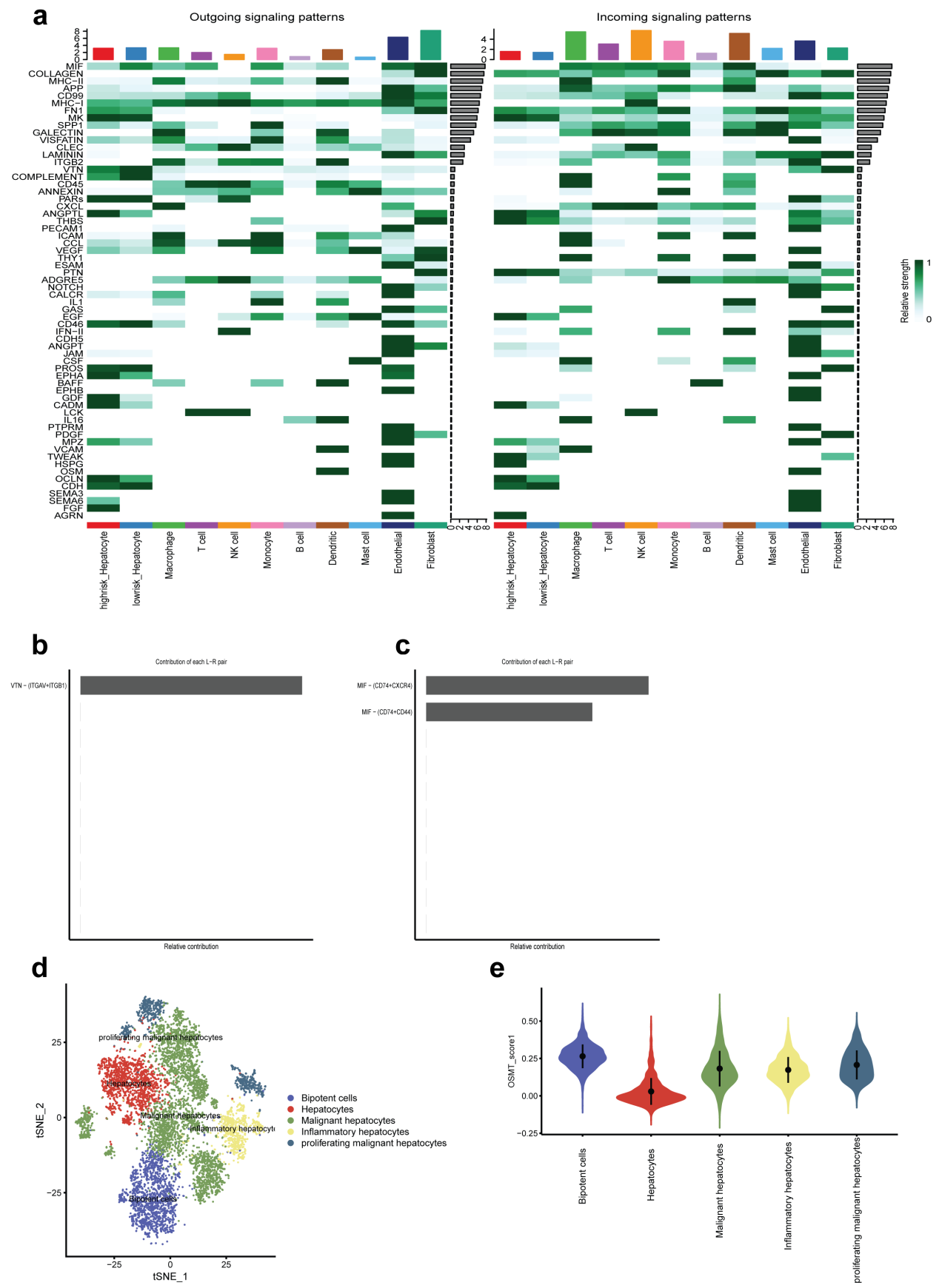


**Fig. S6** Intercellular interactions between high- and low-risk hepatocytes and determination of hepatocytes subtypes. **(a)** Heatmaps represent the outgoing or incoming signaling of certain cell groups. **(b)** The VTN signaling pathway receptor analysis. **(c)** The MIF signaling pathway receptor analysis. **(d)** Clustering of hepatocytes. **(e)** The activity score of OSMT in hepatocytes clusters.

**Supplementary table legends**

**Supplementary table 1**

DEGs between HCC and normal liver tissues (control) in the TCGA-LIHC dataset

**Supplementary table 2**

**Sheet 1** Totals of 1,136 mitochondria-related genes

**Sheet 2** Totals of 1,188 oxidative stress-related genes
